# Cerebral venous sinus thrombosis complicated by seizures: a retrospective analysis of 69 cases

**DOI:** 10.1007/s11239-017-1570-5

**Published:** 2017-10-16

**Authors:** Du-juan Sha, Jian Qian, Shuang-shuang Gu, Lu-na Wang, Fang Wang, Yun Xu

**Affiliations:** 1 Department of Emergency, Nanjing Drum Tower Hospital Affiliated to Nanjing University Medical School, 321 Zhongshan Road, Nanjing, 210008 Jiangsu China; 2 Department of Neurology, Nanjing Drum Tower Hospital Affiliated to Nanjing University Medical School, 321 Zhongshan Road, Nanjing, 210008 Jiangsu China; 3 The State Key Laboratory of Pharmaceutical Biotechnology, Nanjing University, Nanjing, 210008 China

**Keywords:** Sinus thrombosis, Epileptic seizures, Risk factors, Outcomes

## Abstract

Cerebral venous sinus thrombosis (CVST) is a rare ischemic cerebrovascular disease. The aim of this retrospective observational study was to investigate the risk factors for complication of cerebral venous sinus thrombosis by seizures and to explore the impact of such seizures on clinical outcomes. Patients with cerebral venous sinus thrombosis with or without epileptic seizures were retrospectively analyzed and compared in terms of clinical variables, causative factors, clinical presentation, and imaging data. In all, 69 patients with cerebral venous sinus thrombosis were enrolled in this study, 32 (46.38%) of whom had experienced secondary seizures. Compared with those with no seizures, significantly more patients with secondary seizures had hemiplegia (37.50 vs. 15.63%; P = 0.020), bleeding (29.40 vs. 10.81%; P = 0.047), lesions involving the frontal (31.25 vs. 10.81%; P = 0.023) and temporal lobe (43.75 vs. 8.11%; P = 0.005), and thrombosis in the superior sagittal sinus (65.63 vs. 40.54%; P = 0.036). Multivariate logistic regression analysis showed focal neurological deficits (P = 0.004, odds ratio = 5.16, 95% CI 1.99–15.76) and thrombosis of the superior sagittal sinus (P = 0.039, odds ratio = 0.13, 95% CI 0.04–0.37) were independent risk factors for secondary seizures in patients with cerebral venous sinus thrombosis. In addition, mortality rate (9.38 vs. 5.41%; P = 0.469) and 90-day excellent prognosis rate (81.25 vs. 86.47%; P = 0.793) did not differ significantly between patients with and without epileptic seizures. The presence of focal neurological deficits and thrombosis of the superior sagittal sinus are independent risk factors for secondary seizures in patients with cerebral venous sinus thrombosis, whereas mortality and 90-day prognosis have no correlation with secondary seizures.

## Introduction

Cerebral venous sinus thrombosis (CVST), first reported by Ribes in 1825, is a particular form of ischemic cerebrovascular disease and accounts for 0.5–1% of all cerebrovascular diseases [1]. It affects mostly young to middle-aged adults [2] and can be associated with significant morbidity and mortality [3, 4]. Because CVST have a more varied clinical presentation than other stroke types and they are more difficult to recognize in the early stages [5, 6]. Hence it is important to be aware of the varied clinical presentation, for most of these patients have an excellent outcome if treated early and appropriately.

Presenting symptoms include progressive headache and vomiting, focal neurological deficits, epileptic seizures, and varying degrees of disturbance of consciousness and mental symptoms. Epileptic seizures are a common and important clinical manifestation of CVST, occurring in about one-third of them [7–9]. Sidhom et al. reported that in 29% of the patients, seizure was the first sign and of which 59% had a generalized seizure [10]. Secondary epileptic seizures are reportedly an important risk factor for short-term death in patients with CVST [2, 8, 11] and it is particularly important to finding predictors of assess the risk factors for such seizures in these patients. That may help us to decide in whom treatment with prophylactic antiepileptic drugs would be beneficial, therefore reducing the possible harm of seizures. However, few studies concerning the predictors of seizures and their influence on mortality have been conducted up to now [8, 12, 13].We therefore retrospectively analyzed the clinical data of patients who had been diagnosed with CVST. The aim of our study is to investigated the predictors of presenting seizures in patients with CVST and their influence on outcome, which may help neurologists determine under what circumstances initiating antiepileptic drugs treatment is most beneficial.

## Subjects and methods

### Subjects

Patients with CVST admitted to Nanjing Drum Tower Hospital of Nanjing University Medical School from September 2010 to January 2015 were enrolled in this retrospective study. All patients were diagnosed as having CVST by brain MRI and magnetic resonance venography (MRV) in accordance with the 2012 criteria of the American Heart Association/American Stroke Association [1]. The patients were allocated to seizure and non-seizure groups.

The exclusion criteria were as follows: age less than 18 years; cavernous sinus thrombosis; history of migraine, or tension or cluster headaches; contraindications to anticoagulant drugs; intracranial hematoma or intracranial lesions leading to adjacent sinus venous sinus stenosis or occlusion; a history of epilepsy or antiepileptic drug treatment; and severe systemic or neurological disease.

Diagnosis and classification of epileptic seizures was in accordance with the Guidelines for Epidemiologic Studies on Epilepsy formulated by the International League Against Epilepsy in 1993 [14]. Seizures occurring within 7 days of CVST were defined as early onset and those occurring > 7 days after CVST as late-onset epileptic seizures.

### Data collection

Relevant patient variables, medical history and clinical data, including neurological indications of location of lesion, laboratory and imaging findings, and therapeutic procedures, were collected to analyze the risk factors for causes, including local infection, puerperal period, pregnancy, oral contraceptives, protein S deficiency, and systemic lupus erythematosus. Clinical features included fever, headache, vomiting, altered consciousness (Glasgow Coma Scale scores ≤ 8 points being defined as coma), and focal neurologic signs, including paralysis and sensory disturbances. Performance of activities of daily living was assessed according to the Barthel Index [15], ≤ 40 points being defined as poor, 41–60 points as moderate, and > 60 as good. Imaging findings included bleeding, location of lesion, and location of thrombosis.

Except for those who died during hospitalization, all patients had been followed up for at least 90 days.

Correlations between the above-listed characteristics and occurrence of seizures were then assessed and risk factors for epileptic seizures in patients with CVST and their impacts on clinical outcomes explored.

All study subjects had received subcutaneous low molecular weight heparin (nadroparin calcium 0.1 mL/10 kg, once per 12 h) for 14 days, and oral warfarin thereafter as anticoagulant therapy. The dose of warfarin was adjusted according to the international normalized ratio (INR 2–3). Patients with epileptic seizures were given antiepileptic drugs and mannitol to reduce their intracranial pressure.

### Statistical analysis

All data were analyzed using SPSS17.0 statistical software. Measurement data are expressed as mean ± SD and intergroup comparisons were achieved with the independent-sample *t*-test. Enumeration data are expressed as frequency and percentage (%) and their intergroup comparisons were performed with the χ^2^ test. Multivariate logistic regression analysis was employed to identify independent risk factors for secondary seizures in patients with CVST and the odds ratios (OR) and 95% confidence intervals (CI) calculated. P values of < 0.05 were defined as statistically significant.

## Results

### General condition of participants

In all, 69 patients with CVST were enrolled in this study, including 31 men and 38 women aged 22–46 years with a mean age of 34.51 ± 7.42 years. Thirty-two of them (46.38%) had experienced secondary epileptic seizures and 37 had not. Secondary seizures were of early onset in 19/32 cases and late onset in 13/32 cases.

### Risk factors for secondary seizures

**Fig. 1:**
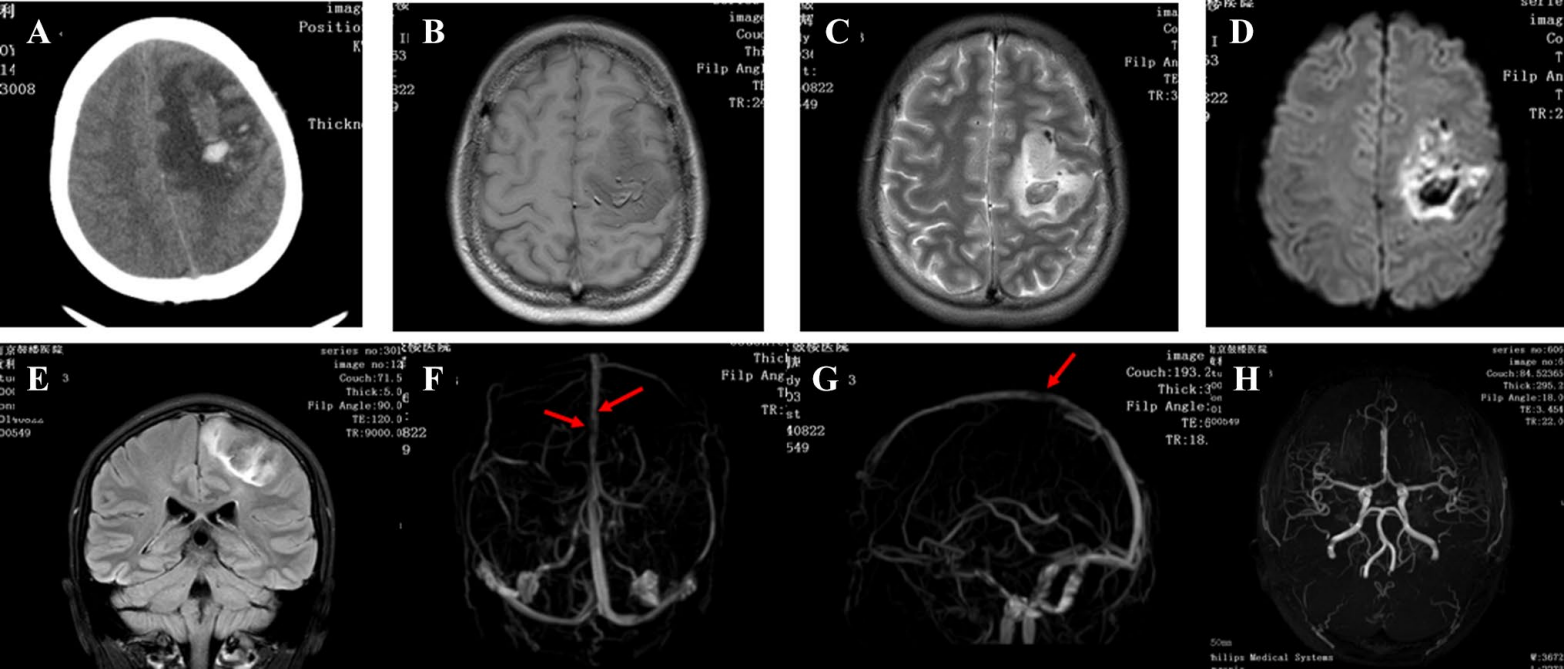
Imaging findings of a representative patient with CVST and concurrent epileptic seizures. **a** Brain CT scan showing left frontal lobe hemorrhage accompanied by surrounding white matter edema. **b** T1-weighted MRI showing left-sided intralesional bleeding. **c** T2-weighted MRI showing intralesional bleeding, edema and mixed signals on the left side. **d** Diffusion-weighted MRI showing mixed high signals. **e** T1-weighted coronal MRI showing mixed signals in the left frontal lobe and edema around the lesions. **f** and **g** MR venography showing filling defects in the sagittal sinus (*red arrows*). **h** MR angiography showing no abnormalities in the intracranial arteries

There were no differences between the non-seizure and seizure groups in age (P = 0.433), sex (P = 0.788) and cause of CVST. Additionally, fever (P = 0.366), headache (P = 0.111), vomiting (P = 0.367) and coma (P = 0.278) were not associated with occurrence of secondary seizures (Table 1). Compared with the non-seizure group, more patients with secondary seizures had focal neurological signs (43.75 vs. 24.32%; χ^2^ = 9.226, P = 0.002), particularly hemiplegia (37.50 vs. 15.63%; χ^2^ = 5.240, P = 0.020); bleeding (29.40 vs. 10.81%; χ^2^ = 3.818, P = 0.047); lesions involving the frontal (31.25 vs. 10.81%; χ^2^ = 5.008, P = 0.023) and temporal lobes (43.75 vs. 8.11%; χ^2^ = 7.318, P = 0.005); and thrombosis in the superior sagittal sinus (65.63 vs. 40.54%; χ^2^ = 4.264, P = 0.036; Table 1; Fig. 1). Lesions involving the parietal (P = 0.498) and occipital lobes (P = 0.231), and thrombosis in the transverse (P = 0.810), sigmoid (P = 0.839) or straight sinuses (P = 0.366) were not associated with occurrence of secondary seizures our study cohort (Table 1; Fig. 1).

**Table 1:** Risk of secondary seizures in patients with cerebral venous sinus thrombosis according to the χ^2^ test

Variable (n, %)	Seizure group (n = 32)	Non-seizure group (n = 37)	χ^2^	P
Gender				
Male	18 (56.25)	22 (59.45)	0.071	0.788
Etiology risk factors				
Local infection	5 (15.63)	7 (18.92)	0.128	0.718
Puerperium	6 (18.75)	5 (13.51)	0.346	0.554
Gestation	3 (9.38)	4 (10.81)	0.038	0.844
Oral contraceptives	7 (21.88)	6 (16.22)	0.354	0.549
Protein S deficiency	1 (3.13)	3 (8.11)	0.769	0.364
Systemic lupus erythematosus	2 (6.25)	3 (8.11)	0.087	0.766
Unknown reason	7 (21.88)	9 (24.32)	0.134	0.712
Clinical manifestations				
Fever	8 (25.00)	6 (16.22)	0.807	0.366
Headache	32 (100.00)	35 (94.59)	1.756	0.111
Vomiting	5 (15.63)	9 (24.32)	0.791	0.367
Coma (GCS ≤ 8)	3 (9.38)	1 (2.70)	1.115	0.278
Focal neurological location signs	14 (43.75)	9 (24.32)	9.226	0.002
Hemiplegia	12 (37.50)	5 (15.63)	5.240	0.020
Sensory disturbances	2 (6.25)	4 (10.81)	0.443	0.498
Imaging findings				
Infarction	4 (12.50)	5 (13.51)	0.015	0.901
Hemorrhage	10 (29.41)	4 (10.81)	3.818	0.047
Thrombosis site				
Superior sagittal sinus	21 (65.63)	15 (40.54)	4.264	0.036
Transverse sinus	7 (21.88)	9 (24.32)	0.057	0.810
Sigmoid sinus	12 (37.50)	13 (35.14)	0.041	0.839
Straight sinus	8 (25.00)	6 (16.22)	0.807	0.366
Lesions involving the site				
Frontal lobe	10 (31.25)	4 (10.81)	5.008	0.023
Temporal lobe	14 (43.75)	3 (8.11)	7.318	0.005
Parietal lobe	2 (6.25)	4 (10.81)	0.443	0.498
Occipital lobe	1 (3.13)	2 (5.41)	1.156	0.231

*GCS* Glasgow Coma Scale

Multivariate logistic regression analysis showed that focal neurological deficits (P = 0.004, OR 5.16, 95% CI 1.99–15.76) and thrombosis of the superior sagittal sinus (P = 0.039, OR 0.13, 95% CI 0.04–0.37) were independent risk factors for secondary seizures in patients with CVST (Table 2).

**Table 2:** Multivariate logistic regression analysis of relevant factors in patients with CVST and secondary seizures

Risk factors	OR	95% CI	P
Headache	1.355	0.346–5.302	0.663
Altered consciousness	0.167	0.019–1.467	0.106
Focal neurological deficits	5.167	1.993–15.764	0.004
Lesion in the frontal lobe	4.900	0.938–25.595	0.060
Lesion in the temporal lobe	3.778	0.908–15.724	0.068
Thrombosis of the sagittal sinus	0.126	0.042–0.370	0.039
Thrombosis of the transverse sinus	0.815	0.290–2.291	0.698
Thrombosis of the sigmoid sinus	0.648	0.294–1.686	0.374
Thrombosis of the straight sinus	0.460	0.158–1.340	0.155

*CVST* Cerebral venous sinus thrombosis, *OR* odds ratio, *CI* confidence interval

### Effect of secondary seizures on patients’ prognosis

Three subjects (9.38%) in the seizure group died; two of them during hospitalization and one after leaving hospital against medical advice, whereas in the non-seizure group, two subjects (5.41%) died, one during hospitalization and one after leaving hospital against medical advice; these differences are not significant (P = 0.469). At 90 days after onset, there were four patients (12.50%) with poor outcomes, two (6.25%) with moderate outcomes, and 26 (81.25%) with good outcomes in the seizure group, whereas in the non-seizure group two cases (5.41%) had poor outcomes, three (8.11%) of moderate outcomes and 32 (86.47%) good outcomes; these differences between the two groups are not significant (P = 0.793; Table 3).

**Table 3:** Outcomes 90 days after onset in patients with CVST according to presence or absence of epileptic seizures

Prognosis (n, %)	Seizure group (n = 32)	Non-seizure group (n = 37)	χ^2^	P
Poor recovery^a^	4 (12.50)	2 (5.41)		
Death	3 (9.38)	2 (5.41)	0.512	0.469
Barthel Index ≤ 40	1 (3.13)	0 (0)		
Moderate recovery	2 (6.25)	3 (8.11)		
Good recovery	26 (81.25)	32 (86.47)	0.346	0.793

^a^There were six cases of poor prognosis in the two groups: in the seizure group, two cases died during hospitalization and one case died after being discharged; in the non-seizure group, one case died during hospitalization and one case died after being discharged

## Discussion

CVST, a rare ischemic cerebrovascular disease with an annual incidence of 3–4/1,000,000 in adults [16–18], is more common in women than men [6, 19] and in the age range of 20–40 years [20]. Good outcomes are reportedly achieved in 79% of patients with CVST, the mortality rate being 9–15% [21, 22] and 1-year recurrence rate 6.5% [23, 24]. In an international multi-center study of 11,400 hospitalized patients with CVST, Nasr et al. reported an average age was 38.1 years [25]. In the present study, female patients comprised 55.07% of all patients and were aged 22–46 years with a mean age of 34.51 ± 7.42 years; these data are consistent with previous findings [22, 23].

Reported studies have reached different conclusions concerning the incidence of epileptic seizures in patients with CVST. Ferro et al. [1] reported that about 40% of patients with CVST develop epileptic seizures whereas in Kalita et al.’s retrospective study, from Kalita et al. [12], 46.70% of patients with CVST developed secondary seizures. Bharatkumar et al. reported that 63.75% (51/80) of patients with CVST complicated by seizures [26].In our cohort, 32/69 patients (46.38%) with CVST had secondary seizures. Gestation and the puerperium are both high risk factors for CVST [11, 27]; however, whether they alter the risk of secondary seizures is controversial. Studies suggested that the risk factors included pregnancy, puerperium period and coagulopathies (10, 30), Gadelha et al. [29] reported that young mothers and women with preeclampsia and CVST have a higher risk of secondary seizures; however, Bertina et al.’s data [30] did not support this conclusion, and nor did ours.

Epileptic seizures are associated with damage to the motor cortex and surrounding cortical tissues. Studies have shown that when CVST is accompanied by intracranial hemorrhage, local movement disorders, and cortical venous sinus thrombosis, epileptic seizures are more likely to develop. CVST occurs most often in the superior sagittal sinus (62%), followed by the transverse (41.2–44.7%) and straight sinuses (18%). In approximately 30% of patients, the CVSTs involve both the superior sagittal and transverse sinuses [21, 23]. The superior sagittal sinus and cortical vein drain blood from the supratentorial parenchyma, especially the motor and sensory cortex. There are few reports on whether secondary seizures following CVST are associated with lesions involving particular regions of the brain [8]. Patients with CVST and secondary epileptic seizures most often have superior sagittal, straight or cortical venous sinus thrombosis, accompanied by hemiplegia, hemidysesthesia and aphasia [8]. A recent study by Davoudi et al. [13] showed that 34% of patients with CVST develop secondary seizures and identified the supratentorial region as the only location independently correlated with development of epileptic seizures (OR 4.67, 95% CI 1.51–15.08, P = 0.005).Moreover, secondary seizures did not increase the risk of death and other poor outcomes in their patients. We found that, compared with the non-seizure group, more patients in the seizure group had hemiplegia, bleeding, lesions involving the frontal and temporal lobes, and superior sagittal sinus thrombosis. Multivariate logistic regression analysis showed focal neurological deficits and superior sagittal sinus thrombosis are both independent risk factors for secondary seizures in patients with CVST. These findings are consistent with those of Ferro et al. [8], indicating that active anticoagulant and antiepileptic treatment is indicated in patients with CVST, focal neurological deficits at onset, and superior sagittal sinus thrombosis on MR images.

Several studies have shown that secondary seizures have no effect on the long-term prognosis of patients with CVST but are an important risk factor for short-term death. Beghi et al. [31] pointed out the mortality of patients with CVST and secondary epileptic seizures (12.5%) was higher than that of those without epileptic seizures (6.3%). However, in the present study, the mortality and 90-day recovery rate did not differ significantly between the seizure and non-seizure groups (9.38 vs. 5.41%, P = 0.469; 81.25 vs. 86.47%, P = 0.793, respectively), which is consistent with the findings of Kalita et al. [12].

In conclusion, we found that focal neurological deficits and superior sagittal sinus thrombosis are both independent risk factors for secondary seizures following CVST and that mortality and 90-day prognosis are not correlated with secondary epileptic seizures. In the future, large prospective studies are needed to assess the risk factors for secondary seizures following CVST.

## Limitations

The sample sizes of our research is small. In the future, large prospective studies are needed to assess the risk factors for secondary seizures following CVST.
